# Prospective pharmacotyping of urothelial carcinoma organoids for drug sensitivity prediction – feasibility and real world experience

**DOI:** 10.1186/s40164-024-00579-3

**Published:** 2024-11-12

**Authors:** Michael Karl Melzer, Yanchun Ma, Jessica Lindenmayer, Clara Morgenstern, Felix Wezel, Friedemann Zengerling, Cagatay Günes, Nadine Therese Gaisa, Alexander Kleger, Christian Bolenz

**Affiliations:** 1 Department of Urology, Ulm University Hospital, Ulm, Germany; 2 Institute for Molecular Oncology and Stem Cell Biology, Ulm University Hospital, Ulm, Germany; 3 Institute of Pathology, Ulm University Hospital, Ulm, Germany; 4 Core Facility Organoids, Ulm University, Ulm, Germany; 5 Section for Interdisciplinary Pancreatology, Clinic for Internal Medicine I, Ulm University Hospital, Ulm, Germany

**Keywords:** Patient-derived organoids, Urothelial carcinoma, Pharmacotyping

## Abstract

Urothelial carcinoma (UC) of the urinary bladder has significant challenges in treatment due to its diverse genetic landscape and variable response to systemic therapy. In recent years, patient-derived organoids (PDOs) emerged as a novel tool to model primary tumors with higher resemblance than conventional 2D cell culture approaches. However, the potential of organoids to predict therapy response in a clinical setting remains to be evaluated. This study explores the clinical feasibility of PDOs for pharmacotyping in UC. Initially, we subjected tumor tissue specimens from 50 patients undergoing transurethral resection or radical cystectomy to organoid propagation, of whom 19 (38%) yielded PDOs suitable for drug sensitivity assessment. Notably, whole transcriptome-based analysis indicated that PDOs may show phenotypes distinct from their parental tumor tissue. Pharmacotyping within a clinically relevant timeframe [mean of 35.44 and 55 days for non-muscle invasive bladder cancer (NMIBC) and muscle invasive bladder cancer (MIBC), respectively] was achieved. Drug sensitivity analyses revealed marked differences between NMIBC and MIBC, with MIBC-derived organoids demonstrating higher chemosensitivity toward clinically relevant drugs. A case study correlating organoid response with patient treatment outcome illustrated the complexity of predicting chemotherapy efficacy, especially considering the rapid acquisition of drug resistance. We propose a workflow of prospective organoid-based pharmacotyping in UC, enabling further translational research and integration of this approach into clinical practice.

**Supplementary Information:**

The online version contains supplementary material available at 10.1186/s40164-024-00579-3.

**To the editor**,

Bladder cancer represents a significant health concern with a rising incidence [1]. While non muscle-invasive bladder cancer (NMIBC) is typically treated by transurethral resection (TURBT) followed by instillation therapies [2], muscle-invasive bladder cancer (MIBC) usually requires radical cystectomy (RC) in combination with perioperative chemotherapy, including the agents cisplatin/gemcitabine/methotrexate/vinblastine/adriamycin [3]. Patient-derived organoids (PDO) from urothelial carcinoma (UC) may provide an adequate method to predict therapy response [4–8]. However, their usage in a real-world clinical-translational scenario still must be evaluated.

Here, we report a single center feasibility experience of prospective organoid pharmacotyping. A total of 104 patients were included (Fig. 1A, B, Supplementary Fig. 1, 2, Supplementary Table 1). Of these, 50 patients yielded sufficient tumor material, however organoid growth was insufficient in 30 cases due to (i) weak growth, (ii) overgrowth by other cell types, or (iii) an early decline in proliferative capacity (Fig. 1B). Consequently, PDOs from 20 patients were subjected to pharmacotyping: 12 men, 8 women, with mean ages of 68.25 and 66.8 years (Fig. 1C). 8 organoid lines were derived from TURBT, 1 from nephroureterectomy, and 11 from RC (Fig. 1D). The tumor stages varied: 10 cases MIBC or nodal involvement, 1 case with stage ypT0 (Fig. 1E). The latter case was excluded from further analysis. Notably, growth properties varied throughout different patients (Fig. 1F). Whole transcriptome profiling for 8 organoid lines and their parental tumors showed that sample type (tumor vs. organoid) contributed 42.69% of the explained variance in PC1, likely due to high expression of stroma- and immune-related genes (Fig. 1G, H) supported by gene set enrichment analysis and cell type deconvolution suggesting a strong contribution of immune and stromal cells to the phenotype of the parental tumor (Fig. 1I, J). When investigating MIBC subtype [9], 5 of the 8 organoid lines showed phenotypic alterations on a transcriptional level (Fig. 1K, Supplementary Figs. 2, 3; Supplementary Tables 3, 4). However, after regression for the different cellular composition (Fig. 1J), organoids mostly demonstrated subtype stability. On the other hand, organoid gene signatures are associated with cell division. Subsequently, PDOs exhibited higher proliferative activity (Fig. 1L, M). Thus, the key differences between tumors and PDOs involve cellular composition and proliferation rate.

**Fig. 1:**
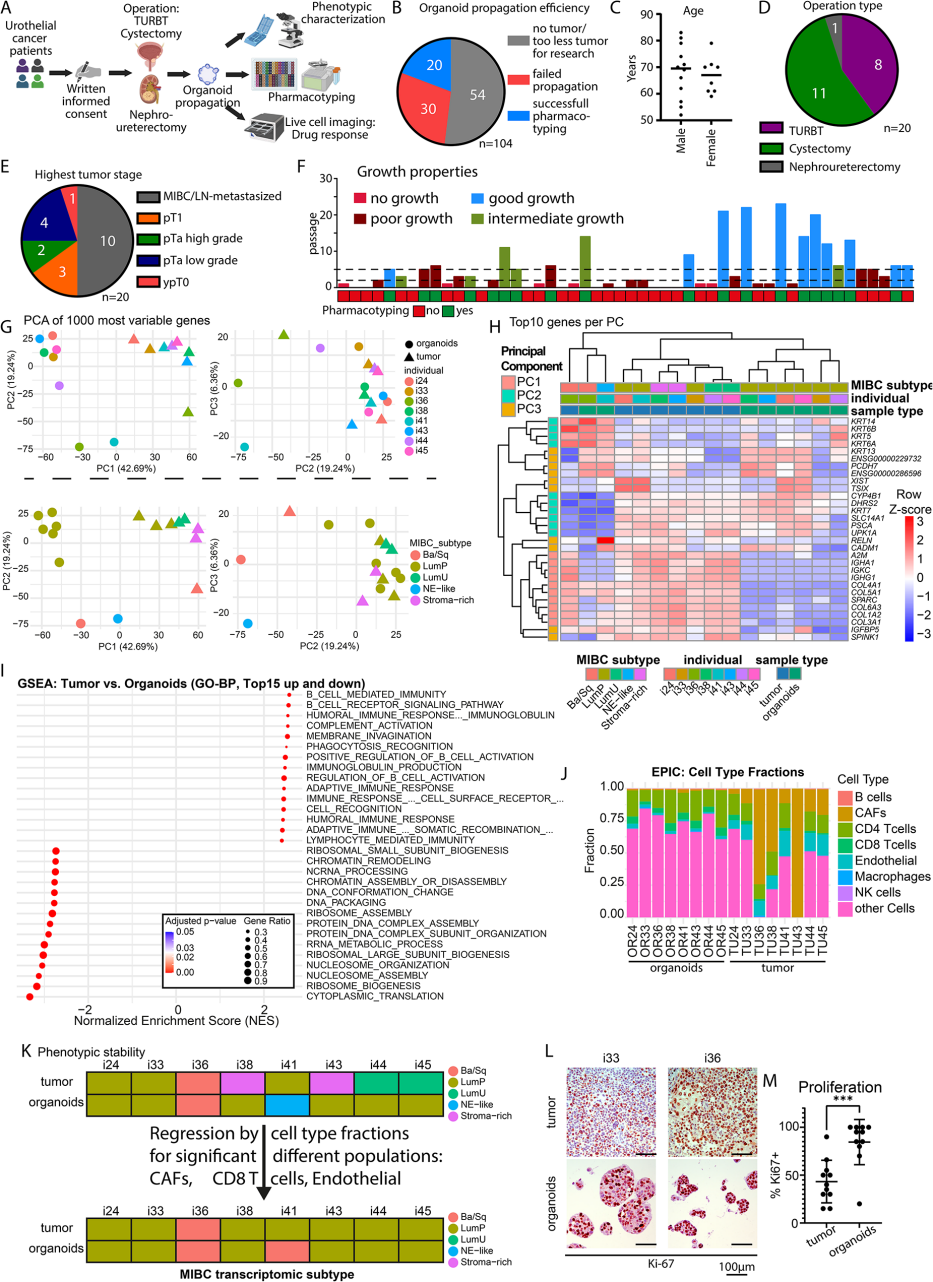
Patient characteristics of recruited patients. (**A**) Schematics of workflow starting at patient recruitment and leading to phenotypic characterization, pharmacotyping, and live cell imaging of propagated organoids. (**B**) Pie chart of recruited patients (*n* = 104). (**C**) Age and sex distribution of pharmacotyped patients (*n* = 20). (**D**) Pie chart for operation type of pharmacotyped patients (*n* = 20). (**E**) Pie chart for highest tumor stage of pharmacotyped patients, assessed by pathological examination (*n* = 20). (**F**) Bar chart of maximum documented passage of organoids (*n* = 50). (**G**) Principal component analysis of the top 1000 most variable genes from indicated organoid and respective tumor samples (*n* = 8). Colors indicate individual in the upper graphs and MIBC subtype in the lower graphs. (**H**) Heatmap of the top 10 genes for the first three principal components. (**I**) Gene set enrichment analysis for the top 15 up and downregulated gene sets in the gene-ontology- biological process dataset (tumor vs. organoids). (**J**) Transcriptome-based cell type deconvolution with the EPIC package in 8 organoid and respective tumor samples. (**K**) Transcriptome-based classification of MIBC subtype before and after regression for cell type composition. (**L**) Representative immune-histology stainings for Ki-67 in the 2 indicated organoid lines with corresponding tumors. Scale bars indicate 100 μm. (**M**) Percentage of Ki-67-positive cells in organoids and tumors. Significance was calculated with a paired t-test (***=*p* < 0.001, *n* = 11). Ba/Sq, basal-squamous, GO-BP, gene-ontology biological process, LN, lymph node, LumP, luminal papillary, LumU, luminal unstable, MIBC, muscle-invasive bladder cancer, NE-like, neuroendocrine like, pTa, papillary tumor, TURBT, transurethral resection of bladder tumor

A key goal of our study was to assess the feasibility of prospective pharmacotyping within a clinically reasonable timeframe (Fig. 2A). For NMIBC, the average time from sample acquisition to drug test results was 35 days (Fig. 2B), while MIBC organoid pharmacotyping averaged 55 days. PDOs were tested with chemotherapeutic agents commonly used for UC (Fig. 2C-I, Supplementary Fig. 4). These agents remain valid options for various treatment settings, despite advances with enfortumab-vedotin/pembrolizumab for advanced UC [10]. MIBC organoids showed higher sensitivity than NMIBC organoids for cisplatin, gemcitabine, and doxorubicin. This was confirmed using the Jenks-Natural-Breaks method recently applied to pancreatic cancer organoids [11, 12] to classify PDOs as resistant, intermediate, or sensitive. Synergistic effects between cisplatin and gemcitabine were observed in 4 of 7 organoid lines (Supplementary Fig. 5). Testing gemcitabine/cisplatin on whole PDOs versus single cells showed no difference in resistance patterns (Supplementary Fig. 6).

**Fig. 2:**
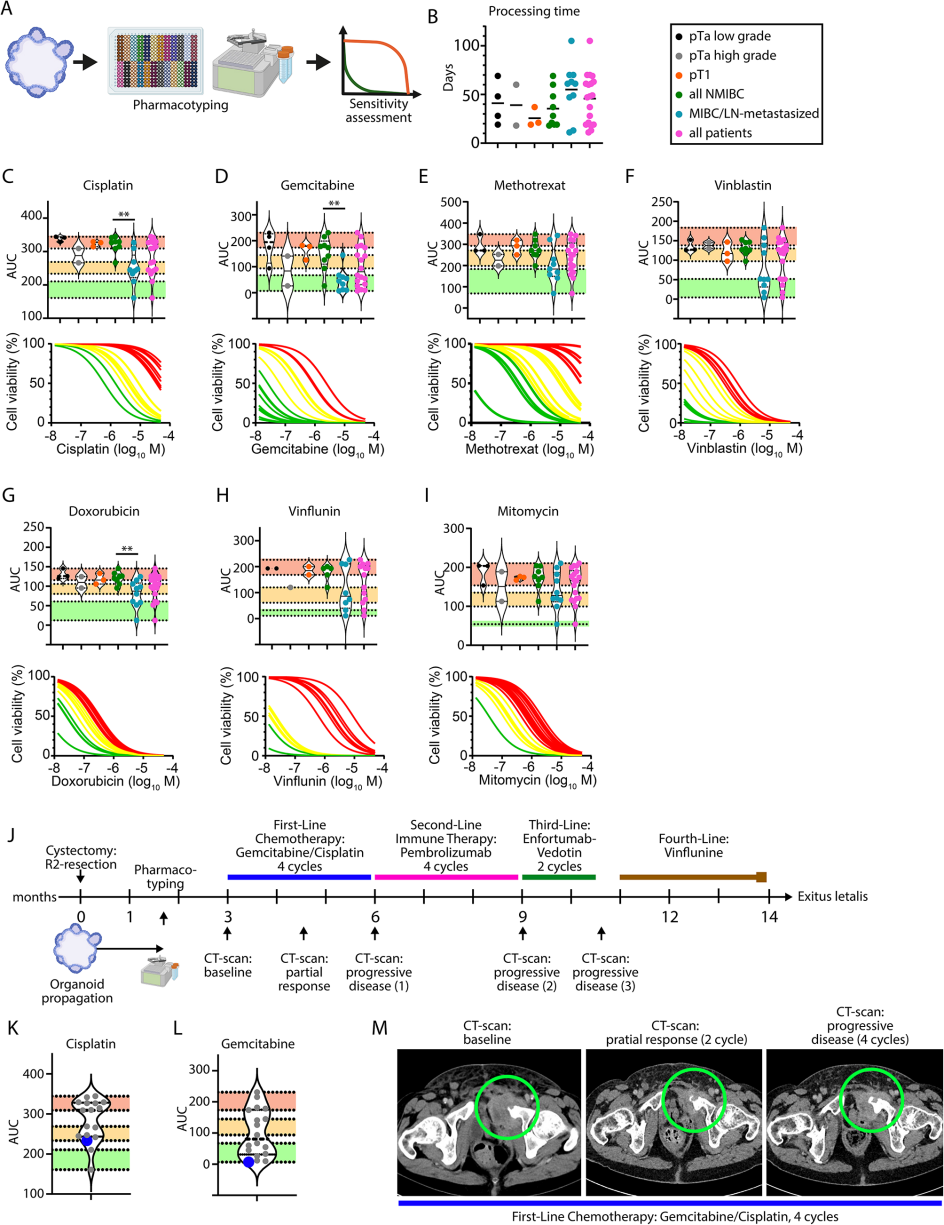
Pharmacotyping of urothelial cancer organoids. (**A**) Schematics of pharmacotyping for urothelial cancer patient-derived organoids. (**B**) Processing time in days of PDOs from sample acquisition to pharmacotyping result depicted for distinct tumor stages and NMIBC, MIBC and all samples (*n* = 19). (**C**) Violinplots for the AUC and dose-response curves for Cisplatin (*n* = 17). (**D**) Violinplots for the AUC and dose-response curves for Gemcitabine (*n* = 18). (**E**) Violinplots for the AUC and dose-response curves for Methotrexate (*n* = 17). (**F**) Violinplots for the AUC and dose-response curves for Vinblastin (*n* = 18). (**G**) Violinplots for the AUC and dose-response curves for Doxorubicin/Adriamycin (*n* = 18). (**H**) Violinplots for the AUC and dose-response curves for Vinflunin (*n* = 13). (**I**) Violinplots for the AUC and dose-response curves for Mitomycin (*n* = 18). (**J**) Schematics of treatment course of the respective patient. (**K**) Violinplot for the AUC values of Cisplatin. The respective patient is highlighted in blue. (**L**) Violinplot for the AUC values of Gemcitabine. The respective patient is highlighted in blue. (**M**) Exemplary CT-scans (venous phase) of the R2-resection area at the symphysis at baseline (before chemotherapy), after 2 and 4 cycles of treatment with gemcitabine/cisplatin. The green circle indicates the tumor mass at the symphysis. Jenks Natural-Breaks method was employed to separate sensitive (green), intermediate (yellow), and resistance (red) PDOs and attribute them to one group. Mann-Whitney test was employed for the comparison of NMIBC and MIBC groups to determine significance levels. *p* < 0.05=*, *p* < 0.01=**. AUC, area under the curve, MIBC, muscle-invasive bladder cancer, NMIBC, non-muscle-invasive bladder cancer

We explored whether pharmacotyping can predict treatment response by tracking a patient post-cystectomy with an R2 resection and early tumor recurrence (Fig. 2J). PDOs indicated an intermediate response to cisplatin and high sensitivity to gemcitabine (Fig. 2K, L). Initial treatment with gemcitabine/cisplatin showed a partial response after two cycles (Fig. 2M). However, all subsequent treatments led to progressive disease and eventually death.

Our study demonstrates that pharmacotyping can be performed within a clinically reasonable timeframe. However, in some cases—particularly for instillation or neoadjuvant therapies—waiting for pharmacotyping results might delay treatment.

A significant challenge in mid-to-long-term organoid propagation is the high dropout rate and phenotypic plasticity compared to the parental tumor—possibly influenced by cell type composition in parental tumors or culture conditions in vitro, but also observed by other groups who focused on short-term pharmacotyping [7, 8]. This underscores the need for further optimization and standardization of organoid culture methods and different protocols.

While our correlation with a patient’s treatment response hints at the promising predictive potential of organoid pharmacotyping for first-line therapy, it also highlights difficulties in capturing tumor plasticity under therapeutic pressure. More extensive correlations with extended patient outcome measures are mandatory to validate the predictive accuracy of PDOs.

## Electronic supplementary material

Below is the link to the electronic supplementary material.

Supplementary Material 1

Supplementary Material 2

Supplementary Material 3

## Data Availability

The datasets generated and/or analysed during the current study are available from the corresponding author on reasonable request. Sequencing data are deposited in the GEO repository (GSE280749).
