# Supplementary material for: Prospective pharmacotyping of urothelial carcinoma organoids for drug sensitivity prediction – feasibility and real world experience
Source: Exp Hematol Oncol. 2024 Nov 12;13:112. doi: 10.1186/s40164-024-00579-3 (PMC11558855; doi:10.1186/s40164-024-00579-3)
Supplement: Supplementary file 2 — Supplementary Material 2 [file 40164_2024_579_MOESM2_ESM.docx]

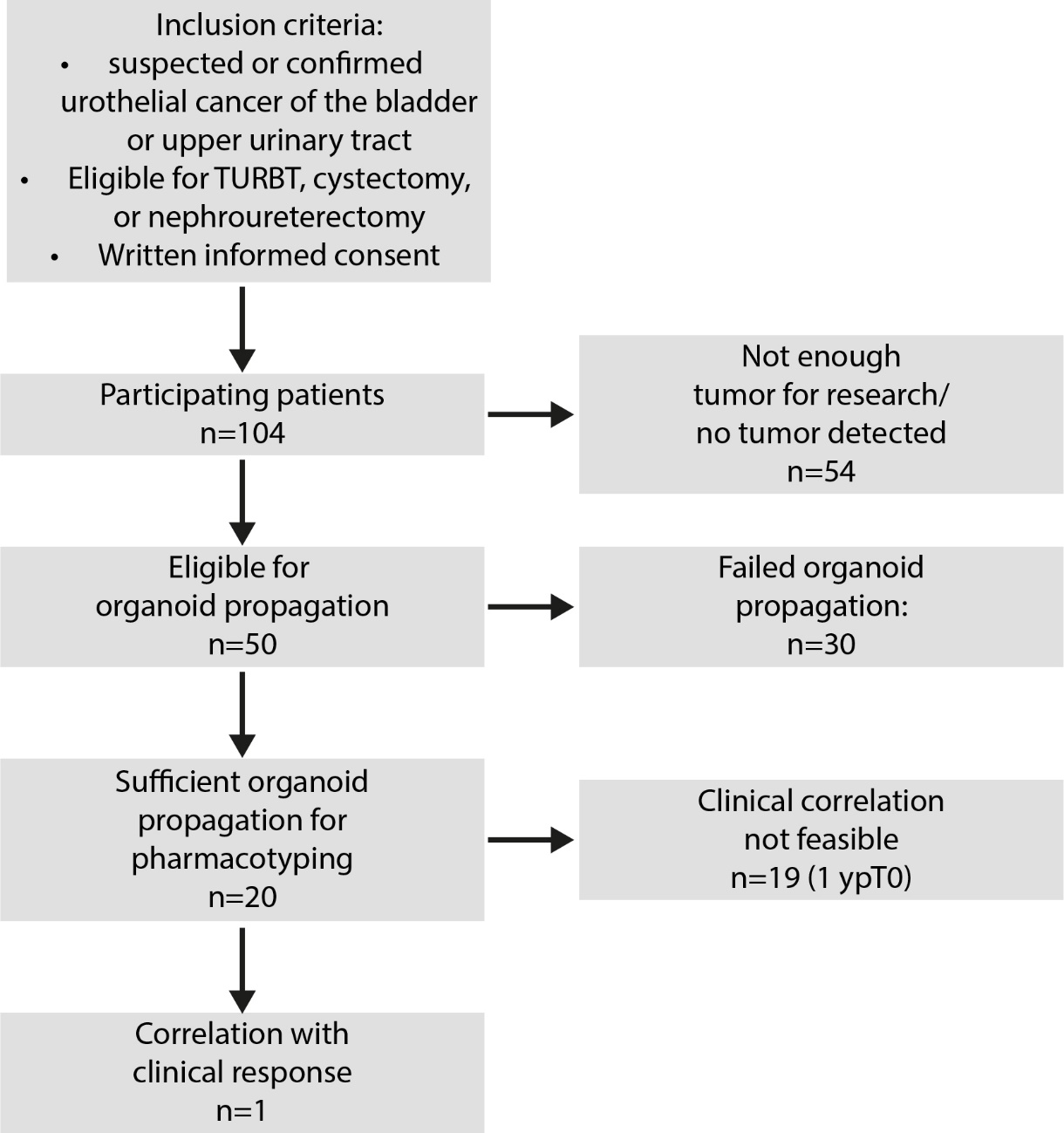


**Supplementary Figure 1: Diagram of patient recruitment**


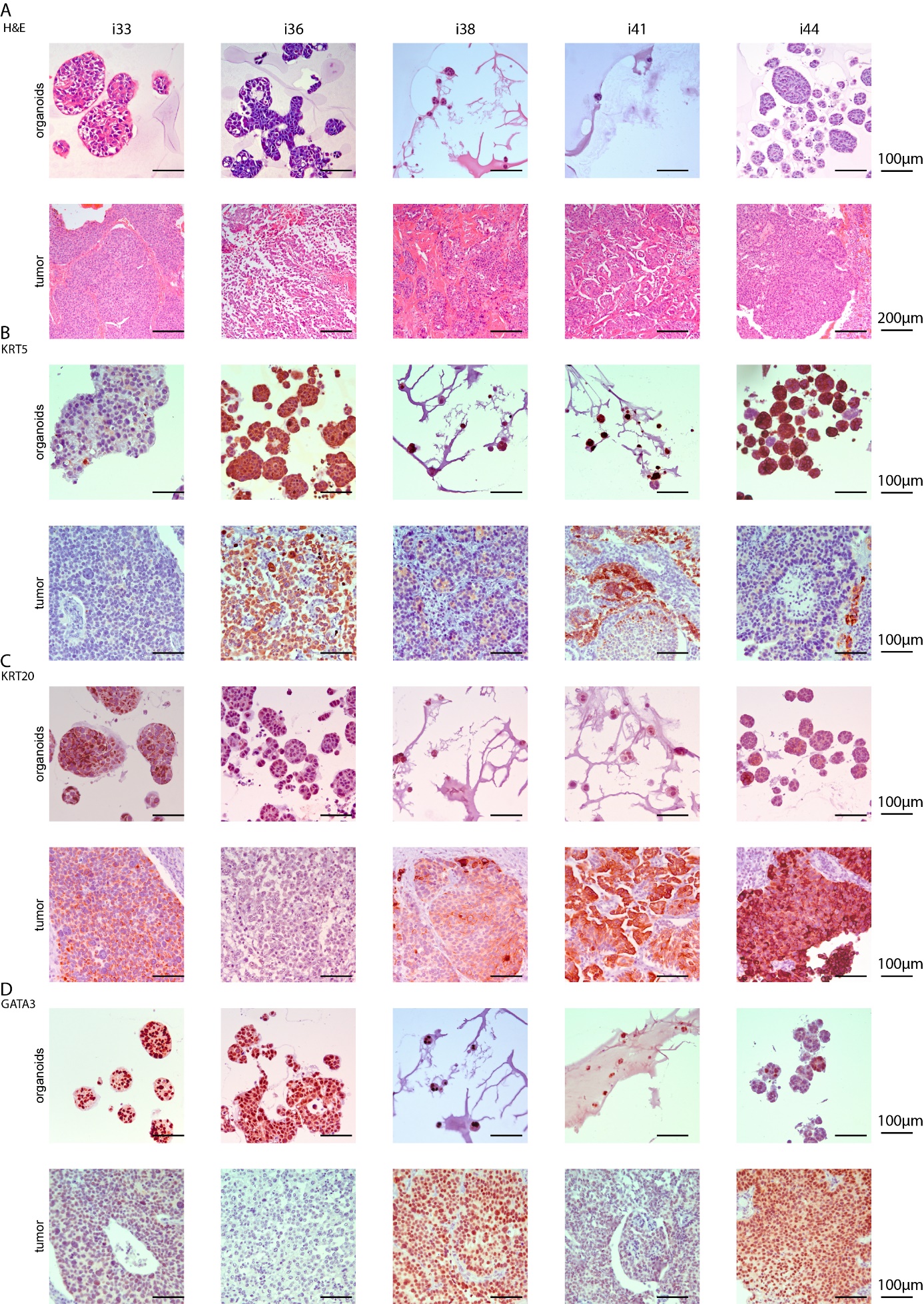


**Supplementary Figure 2: Histological marker expression analysis of representative specimen from the whole transcriptome analysis**

1. H&E staining of the indicated individuals (organoids and tumors). Scale bar presents 100µm for organoids and 200µm for tumors. (n=5)
2. KRT5 staining of the indicated individuals (organoids and tumors). Scale bar presents 100µm. (n=5)
3. KRT20 staining of the indicated individuals (organoids and tumors). Scale bar presents 100µm. (n=5)
4. GATA3 staining of the indicated individuals (organoids and tumors). Scale bar presents 100µm. (n=5)


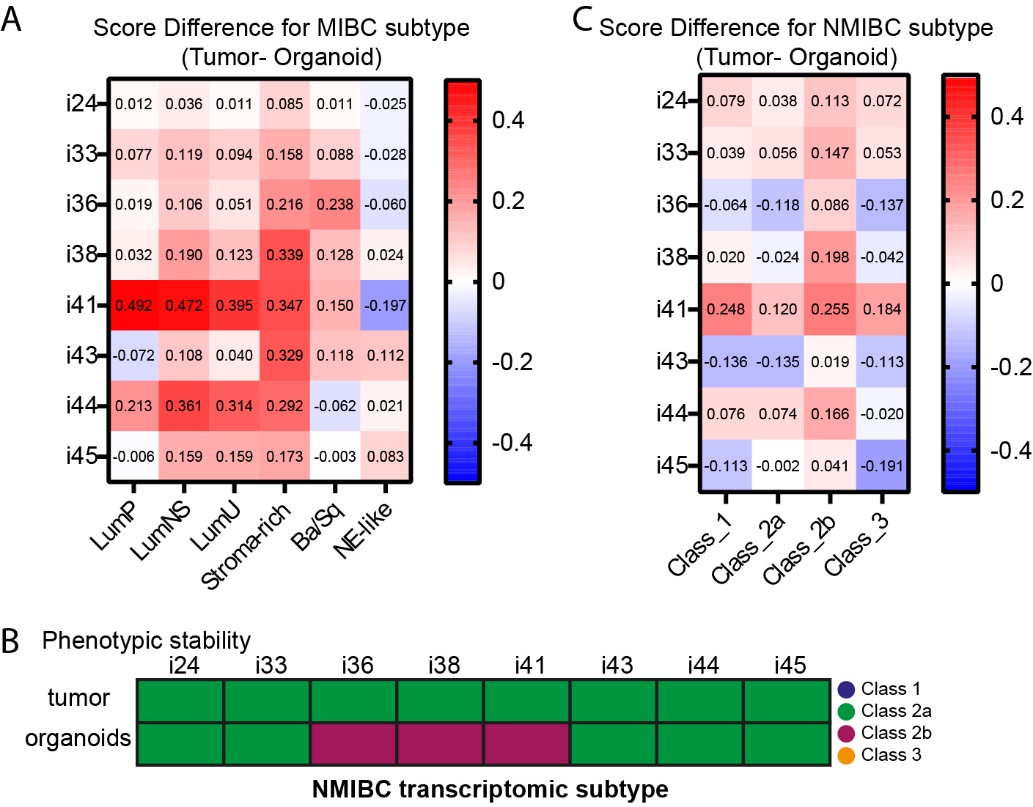


**Supplementary Figure 3: MIBC and NMIBC subtype delineation**

1. Heatmap of absolute subtype score differences for the MIBC subtype scores between tumors and organoids for the indicated individuals. Scale presents positive differences in red, negative differences in blue. (n=8)
2. Graphical illustration of NMIBC subtypes for the whole transcriptome analysis of the indicated individuals. (n=8)
3. Heatmap of absolute subtype score differences for the NMIBC subtype scores between tumors and organoids for the indicated individuals. Scale presents positive differences in red, negative differences in blue. (n=8)

Ba/Sq, basal-squamous, LumNS, luminal non-specified LumP, luminal papillary, LumU, luminal unstable, MIBC, muscle-invasive bladder cancer, NE-like, neuroendocrine like, NMIBC, non muscle-invasive bladder cancer.


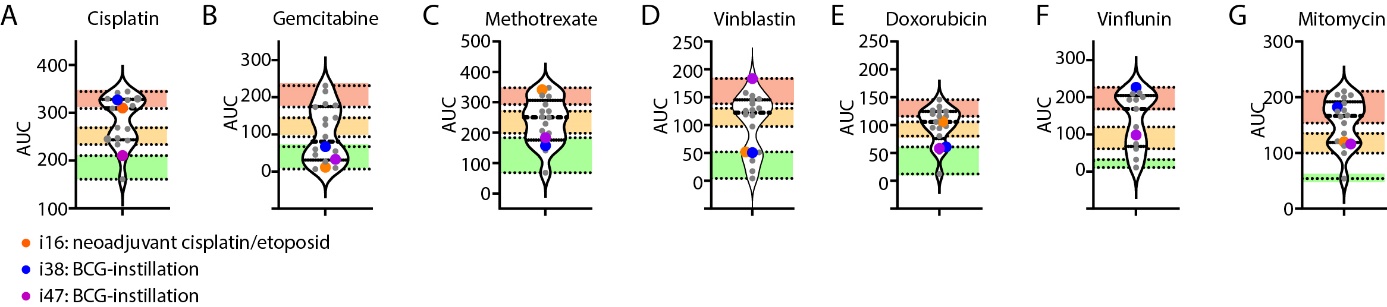


**Supplementary Figure 4: Sensitivity profiles of neoadjuvant treated patients.**

Sensitivity profiles were derived from the same data as in main Figure 2. Patients undergoing previous treatment are highlighted in different colors (i16- orange: neoadjuvant cisplatin/etoposide, i38- blue: BCG-instillation, i47- purple: BCG-instillation. Violin plots are presented for the AUC values of (A) Cisplatin (n=17), (B) Gemcitabine (n=18), (C) Methotrexate (n=17), (D) Vinblastin (n=18), (E) Doxorubicin/Adriamycin (n=18), (F) Vinflunine (n=13), (G) Mitomycin (n=18).


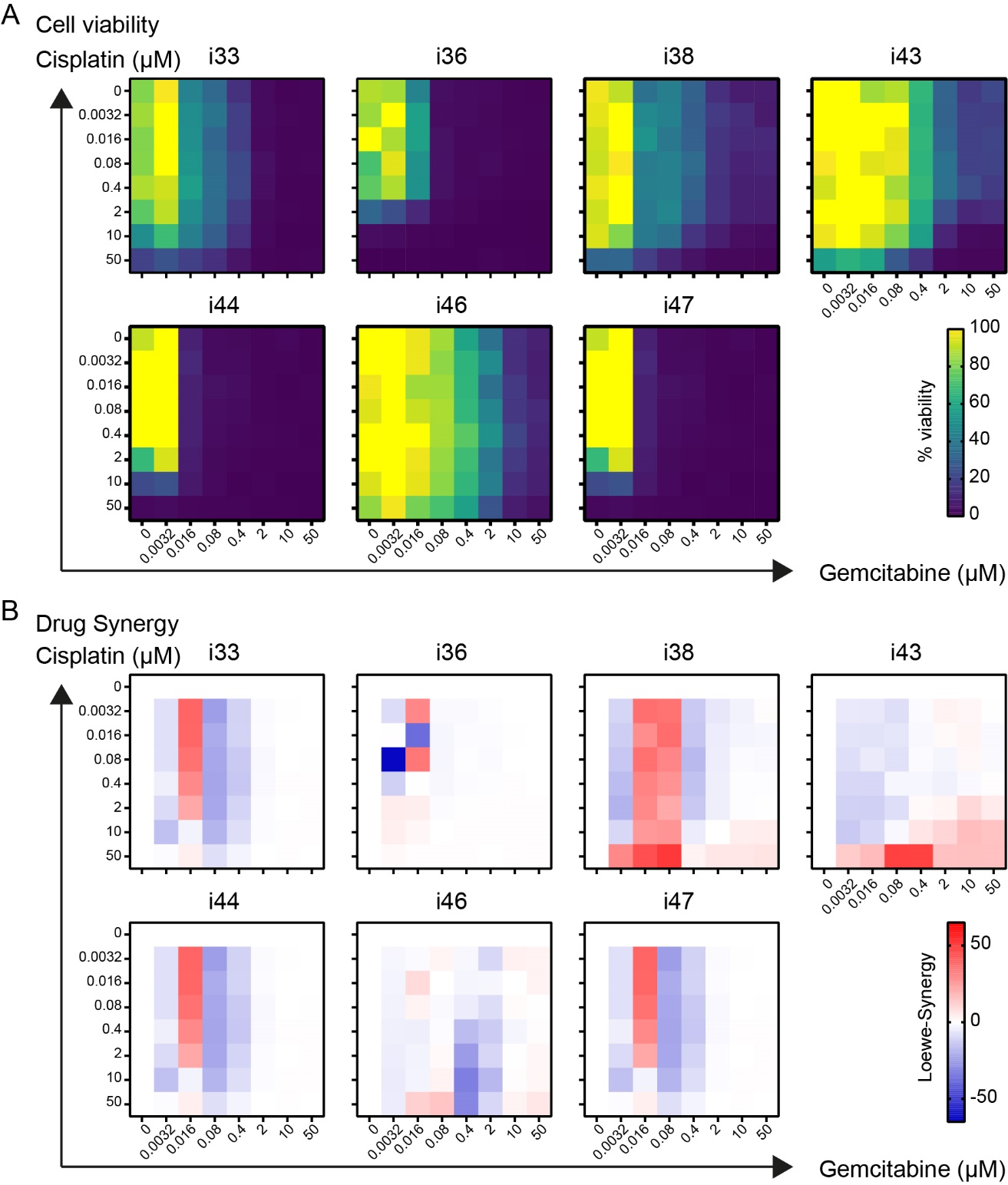


**Supplementary Figure 5: Synergy of gemcitabine and cisplatin in organoids**

1. Heatmaps of cell viability after application of combinations of cisplatin and gemcitabine. x-axis indicates gemcitabine concentration in µM, y-axis indicates cisplatin concentration in µM. Viability is expressed as percentage living cells on a viridis color scale. (n=7)
2. Heatmaps of drug synergism calculated in the SynergyFinder employing the Loewe synergy model. x-axis indicates gemcitabine concentration in µM, y-axis indicates cisplatin concentration in µM. Red indicates synergistic effects, blue antagonistic effects. (n=7)


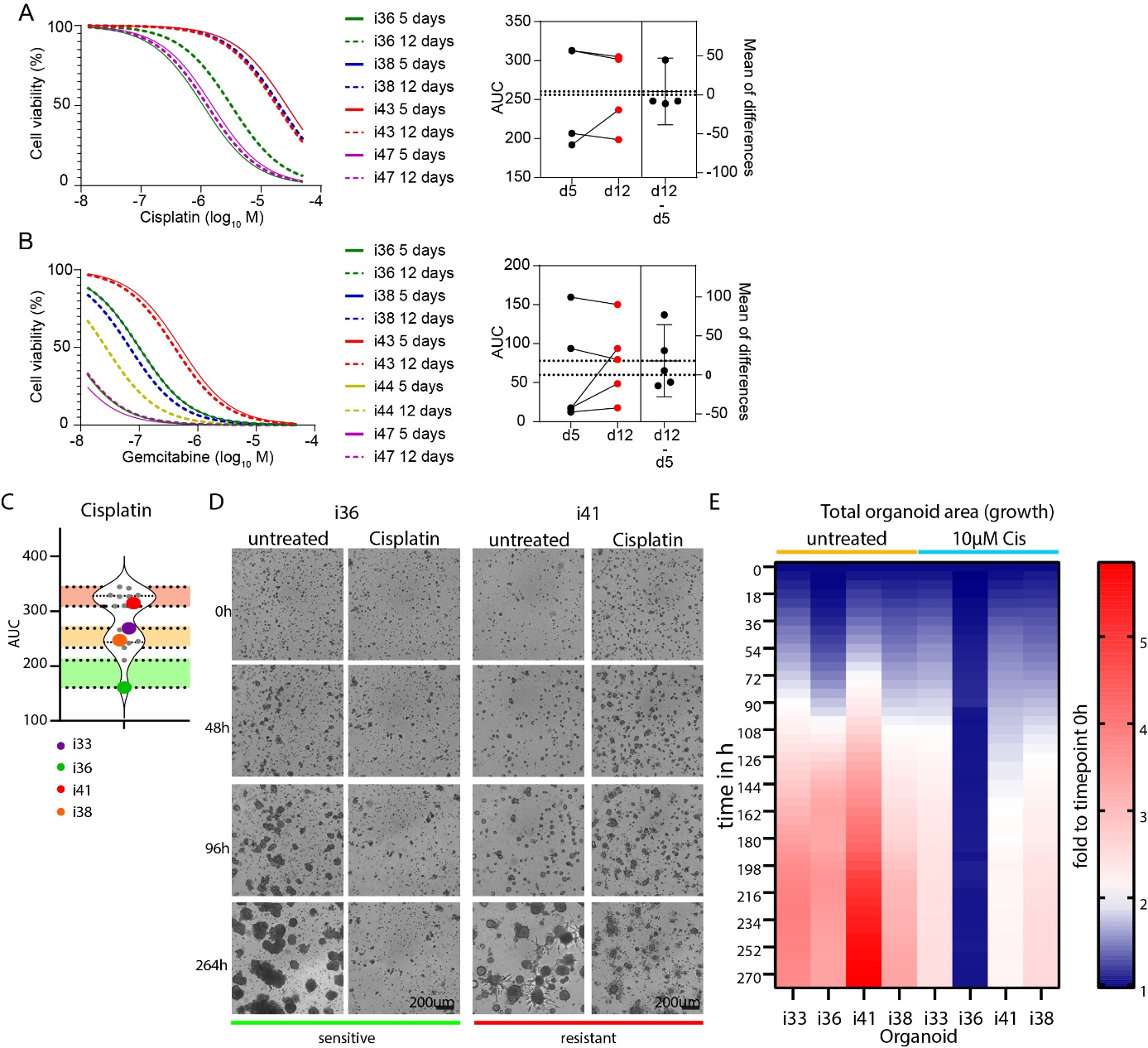


**Supplementary Figure 6: Impact of long-term drug treatment on organoid viability**

1. Dose response profiles of the indicated individuals at the indicated timepoints (12 days vs. 5 days) for cisplatin depicted as dose-response graph and depicted as changes of the AUC. (n=4)
2. Dose response profiles of the indicated individuals at the indicated timepoints (12 days vs. 5 days) for gemcitabine depicted as dose-response graph and depicted as changes of the AUC. (n=5)
3. Violinplot for the AUC of Cisplatin highlighting the PDO lines further subjected to live cell image analysis, as indicated by color (n=17).
4. Representative time-course images at indicated timepoints of one sensitive-classified and one resistant PDO line in response to 10µM Cisplatin or untreated control condition. Scale bar indicates 200µm.
5. Heatmap of normalized growth pattern of the investigated PDO lines in response to 10µM Cisplatin or untreated control condition. (n=4)
