# Supplementary material for: Prospective pharmacotyping of urothelial carcinoma organoids for drug sensitivity prediction – feasibility and real world experience
Source: Exp Hematol Oncol. 2024 Nov 12;13:112. doi: 10.1186/s40164-024-00579-3 (PMC11558855; doi:10.1186/s40164-024-00579-3)
Supplement: Supplementary file 3 — Supplementary Material 3 [file 40164_2024_579_MOESM3_ESM.docx]

**Methods:**

**Patient inclusion, ethics, and tissue acquisition:**

Patients with a presumed or confirmed diagnosis of bladder-confined urothelial cancer were eligible for the prospective recruitment within this study at Ulm University Hospital. Patients underwent either TURBT, radial cystectomy, or nephroureterectomy independently of trial participation. Pieces of tumor tissue that were irrelevant for routine pathological examination were identified by a trained surgeon or pathologist and immediately subjected (<1 h) to organoid propagation. Exclusion criteria for participation in this study included the inability to provide written informed consent or lack of sufficient tumor tissue for research purposes. Patients were enrolled between 2022 and 2023. The project was approved by the local ethics committee (project number 239/18), and written informed consent was obtained from all patients.

**Organoid propagation:**

Urothelial organoid propagation followed a published protocol with some modifications ^1^. Briefly, tumor tissue was rinsed with PBS before being sectioned into approximately 1-2 mm pieces. Subsequently, tumor pieces were digested with Collagenase-II-solution (5 mg/mL in Advanced-DMEM-F12, cat.: 17101015 and 12634010, both Gibco) including Y-27632 (10 µM, cat.: S1049, Selleckchem), GlutaMax, and 10mM HEPES for 1 h at 37°C before further processing. Alternatively, tumor tissue pieces were digested using the human tumor cell dissociation kit (cat.: 130-095-929, Miltenyi) following the manufacturer’s instructions on a gentleMACS Octo Dissociator with Heaters platform (cat.: 130-096-427, Miltenyi) using the 37C_h_TDK_3 program. After neutralization with 10% FCS in DMEM-F12 and centrifugation, remaining red blood cells were lysed in an ACK lysis buffer (0.15 M ammonium chloride, 0.01 M potassium bicarbonate, 0.0001 M disodium EDTA) for 5 min at RT. Cells were counted and up to 2,000,000 cells/mL were resuspended in Ultimatrix (cat.: BME001, Bio-techne) or GFR-Matrigel (cat.: 354230, Corning) to generate 50 µL domes for subsequent culture. After solidification a recently published medium composition ^1^ was used for any further culture. In case, tumors were suspected to have a large stromal content or many dead cells (e.g., due to resection technique of TURBT) became evident during counting a MACS sorting for EpCAM was performed before culture. Briefly, digested tumor pieces were blocked in 10% FCS in DMEM-F12 with Y-27632 for 15-30 min on ice. Further, a mouse EpCAM-APC antibody (1:20 dilution, cat.: 347200, BD Biosciences) was applied in 10% FCS with Y-27632 for 30 min on ice in the dark. Afterwards Anti-Mouse IgG Microbeads (cat.: 130-048-402, Miltenyi) and subsequent sorting on an MS column (cat.: 130-042-201, Miltenyi) were employed following the manufacturer’s instruction for cell sorting using MACS technology. Subsequently, organoid culture was performed as described above. Splitting and singularization of organoid cultures was performed as recently described in detail for our pluripotent stem cell-derived organoid models employing collagenase/dispase digestion followed by accutase treatment ^2,3^.

**Pharmacotyping:**

Pharmacotyping was performed as recently described ^4^ with modifications. Briefly, when reaching enough organoids for pharmacotyping, cultures were singularized. 500 cells per well were seeded into 384-well plates (cat.: 781687, BRAND) using a MANTIS pipetting robot (Formulatrix) in 1µL Matrigel/Ultimatrix and 25 µL of culture medium per well was applied after matrix was polimerized. The following day, organoids were treated with chemotherapeutics by adding another 25 µL of media with the respective drugs. Chemotherapeutics were tested in triplicates using ten concentrations covering a range from 13 nM to 50 µM while only the highest concentration was applied for the solvent controls (DMSO, NaCl), using at least 10 wells per solvent. Incubation was performed for 4 days. Subsequently, cell viability was measured using the CellTiter-Glo® 3D Cell Viability Assay (cat.: G9681, Promega). Luminescence was recorded using a Tecan Infinite M200 Pro (Tecan) and viability is expressed as percentage compared to the mean of the respective solvent control. The area under the curve (AUC) was estimated using the trapezoidal rule with GraphPad Prism v10 (GraphPad Software). The following substances were used: cisplatin (cat.: S1166, Selleckchem, stock 5 mM in NaCl), gemcitabine (cat.: S1714, Selleckchem, stock: 50 mM in DMSO), methotrexate (Medac, 5 mg/mL+25 mg/mL solution), adriamycin/doxorubicin (Medac, 2 mg/mL solution), vinblastine (Teva, 1 mg/mL solution), vinflunine (Pierre Fabre (Javlor), 25 mg/mL solution) and mitomycin (Medac, 1 mg/mL solution). With the exception of cisplatin and gemcitabine, the substances were obtained from the pharmacy of Ulm University Hospital.

**Whole Organoid pharmacotpying:**

Whole organoid pharmacotyping was conducted to assess the impact of drugs on entire organoids rather than individual cells, following a protocol similar to standard pharmacotyping. Specifically, organoids were seeded, and treatment for the control group began one day later, using the same concentrations as previously described. Cytotoxicity was measured five days after seeding. The whole organoid group, seeded as single cells alongside the control group, received treatment starting eight days after seeding, with cytotoxicity assessed on day 12. Treatment was administered over a period of four days.

**Live Cell Imaging:**

For live cell continuous imaging, 10,000 singularized cells/well were seeded into a 96-well plate (cat.: 3595, Corning) in a sandwich format. Plates were precoated with 50 µL 80% Ultimatrix in DMEM-F12. Medium contained 5% Ultimatrix for the seeding. 48 h after seeding and 24 h after starting live cell imaging cells were either treated with 10 µM Cisplatin or left untreated in quadruplicates. Live cell imaging was performed in an Incucyte S3 Live Cell Analysis Instrument (Sartorius) using the multispheroid module with imaging intervals of 6 h. Subsequently, the overall area covered by organoids was used as surrogate parameter to determine the sensitivity to cisplatin. Imaging after treatment was performed for 10 days, medium was changed twice a week.

**Synergy testing:**

Pharmacotyping for synergy tests was carried out in a manner similar to standard pharmacotyping, with slight modifications. Cisplatin and gemcitabine were tested in combination at seven different concentrations, ranging from 0.0032µM to 50µM, as shown in the figures. Incubation lasted for four days, and cell viability was measured as previously described. Synergy scores were then calculated using SynergyFinder 3.0 (available online at <https://synergyfinder.fimm.fi/>)^5^, based on the Loewe additivity synergy model. Positive deviations from expected responses indicate synergy, while negative deviations reflect antagonism.

**Processing of organoids for histology:**

Organoids and primary tissue were processed for histology as recently described in detail ^2,3^. Briefly, organoids were fixed in 4% PFA with 100 mM Sucrose overnight before being embedded in 2% agarose in PBS. Primary tissue was fixed in 3.7% formaldehyde solution. Subsequently the organoids and tissue underwent a standard automated dehydration series before being embedded in paraffin. 4 µm thick sections were prepared before performing histological stainings.

**Histology – Staining, antibodies, analysis:**

Hematoxylin & Eosin (H&E) staining followed recently described standard protocols. Immunostaining for organoids and primary tumor tissue was performed as described recently ^2,3^. The following antibodies and dilutions were used for IHC: GATA3 (1:500, Proteintech Cat# 66400-1-Ig, RRID:AB_2881774), KRT5(1:2000, Proteintech Cat# 66727-1-Ig, RRID:AB_2882077), KRT20 (1:1000, Proteintech Cat# 60183-1-Ig, RRID:AB_10858399), Ki-67 (1:6000, Proteintech Cat# 27309-1-AP, RRID:AB_2756525). Bright-field images were acquired using a Leica DM5500B microscope (Leica) equipped with a Leica DMC5400 camera and Leica Application Suite software (Leica) or by using a Zeiss Axioscope2 microscope (Carl Zeiss) ZEN3.1 imaging software (Carl Zeiss). Modifications for brightness and contrast were only made for illustrative purposes.

**RNA isolation:**

Total RNA from organoids was extracted using the Monarch Total RNA Miniprep Kit (NEB, cat.: T2010S) following the manufacturer’s protocol. RNA from parental tumors was extracted from FFPE tissue using the RNEasy FFPE Kit (Qiagen, cat. 73504), also according to the manufacturer’s guidelines. In brief, tumor regions on slides were identified and marked by a board-certified pathologist, then scraped off the slides. Tissue from multiple sections, with a combined thickness of up to 40µm, was pooled before initiating RNA isolation as per the manufacturer's instructions.

**Library preparation and sequencing:**

The amount of total RNA was quantified using the Qubit 2.0 Fluorometric Quantitation system (Thermo Fisher Scientific, Waltham, MA, USA) and the RNA integrity number (RIN) was determined using the 2100 Bioanalyzer instrument (Agilent, Santa Clara, CA, USA). RNA-seq libraries were prepared with the Watch-maker RNA Library Prep Kit with Polaris™ Depletion (Watchmaker Genomics, Boulder, CO, USA). NGS library concentrations were quantified with the Qubit 2.0 Fluorometric Quantitation system (Life Technologies, Carlsbad, CA, USA) and the size distribution was assessed using the 2100 Bioanalyzer instrument (Agilent, Santa Clara, CA, USA). For sequencing, samples were diluted and pooled into multiplex NGS libraries in equimolar amounts. Expression profiling libraries were sequenced on a NovaSeq 6000 instrument (Illumina, San Diego, CA, USA) following a 50-base-pair, paired-end recipe.

**Processing of RNAseq data:**

NGS reads were mapped to the Genome Reference Consortium GRCh38 assembly via “Spliced Transcripts Alignment to a Reference” (STAR, 2.7.11b) ^6^ utilising the “basic” GENCODE ^7^ transcript annotation from version v46 (May 2024) as reference transcriptome. Since the hg38 assembly flavour of the UCSC Genome Browser was preferred for downstream data processing with Bioconductor packages for entirely technical reasons, GENCODE transcript annotation had to be adjusted to UCSC Genome Browser sequence region names. STAR was run with options recommended by the ENCODE project. NGS read alignments overlapping Ensembl exon features were counted with the Bioconductor (3.18.1) GenomicAlignments (1.36.0) package via the summarizeOverlaps function in Union mode, ignoring secondary alignments and alignments not passing vendor quality filtering. Since dUTP-based RNA-seq protocols lead to the sequencing of the first strand, all alignments needed inverting before strand-specific counting in feature (i.e., gene, transcript, and exon) orientation. Exon-level counts were aggregated to gene-level counts and the Bioconductor DESeq2 (1.40.2)^8^ package was used to test for differential expression based on a model using the negative binomial distribution.

**Analysis and visualization of RNAseq data:**

Data analysis and visualization were conducted using RStudio (version 2023.09.1) with R version 4.3.1. Raw gene counts were used for further analysis, beginning with the removal of genes with a total count of less than 50 across all samples. Next, gene counts were transformed using DESeq2 (version 1.42.1) ^8^ based on a "tumor vs. organoids" design. Following this, variance-stabilizing transformation was applied. Principal component analysis (PCA) was then performed on the top 1000 most variable genes, with visualization generated via the ggplot2 package (version 3.5.1)^9,10^. The top 10 genes for the first three principal components (PCs) were extracted, and gene names were converted from Ensembl-IDs to HGNC symbols using the biomaRt package (version 2.58.2)^11,12^. Heatmaps were created from variance-stabilized counts using the pheatmap package (version 1.0.12). Subtype scores for MIBC and NMIBC were computed from variance-stabilized expression values using the consensusMIBC (version 1.1.0)^13^ and classifyNMIBC (version 1.1.0)^14^ packages, respectively.

Cell type deconvolution and percentage attribution from bulk RNA-seq data were performed on normalized counts using the EPIC package (version 1.1.7)^15^. Limma package (3.58.1) ^16^ with the “removeBatchEffect” function was employed to perform regression of variance stabilized data by significantly different cell type fractions before calculation of subtype scores on regressed data. Significantly different cell type fractions were determined by Wilcoxon test on centered and scaled cell type fractions.

Gene set enrichment analysis (GSEA) was conducted in RStudio using raw counts. After applying DESeq2 for transformation, log2-fold changes were adjusted using the ashr package (version 2.2.63) ^17^. Genes with a baseMean of less than 20 were filtered out. The msigdbr (version 7.5.1) ^18^, clusterProfiler (version 4.10.1) ^19^, BiocParallel (version 1.36.0) ^20^, and dplyr (version 1.1.4) ^21^ packages were then utilized to analyze gene ontology (GO-biological process) datasets. The top 15 up- and down-regulated datasets were visualized as dot plots using the ggplot2 package.

Code writing in RStudio was assisted by OpenAI's GPT-4 language model, via the R Wizard assistant.

Data are deposited in the GEO repository (GSE280749).

**Statistical analysis:**

GraphPad Prism v10 was used for statistical analysis and graphical illustration. Significance levels for pharmacotyping were calculated only for the NMIBC vs MIBC comparison using a Mann-Whitney-U test. Significance levels are indicated in the figures or the respective legends. The Jenks Natural Breaks analysis for this paper was generated using the Real Statistics Resource Pack software (Release 8.9.1). Copyright (2013 – 2023) Charles Zaiontz. www.real-statistics.com. The Jenks-Natural Breaks method was employed ^4,22^ to minimize differences within one group and maximize differences between groups. Dose response curves are depicted as non-linear fit regression model of actual dose response curves.

**Manuscript preparation:**

Writing of the manuscript was assisted by ChatGPT, Version 4, (OpenAI).

1 Mullenders, J. *et al.* Mouse and human urothelial cancer organoids: A tool for bladder cancer research. *Proceedings of the National Academy of Sciences of the United States of America* (2019). <https://doi.org:10.1073/pnas.1803595116>

2 Breunig, M. *et al.* Modeling plasticity and dysplasia of pancreatic ductal organoids derived from human pluripotent stem cells. *Cell Stem Cell* **28**, 1105-1124.e1119 (2021). <https://doi.org:10.1016/j.stem.2021.03.005>

3 Breunig, M. *et al.* Differentiation of human pluripotent stem cells into pancreatic duct-like organoids. *STAR Protocols* **2**, 100913-100913 (2021). <https://doi.org:10.1016/J.XPRO.2021.100913>

4 Beutel, A. K. *et al.* A prospective feasibility trial to challenge patient–derived pancreatic cancer organoids in predicting treatment response. *Cancers* **13** (2021). <https://doi.org:10.3390/cancers13112539>

5 Ianevski, A., Giri, A. K. & Aittokallio, T. SynergyFinder 3.0: an interactive analysis and consensus interpretation of multi-drug synergies across multiple samples. *Nucleic Acids Res* **50**, W739-w743 (2022). <https://doi.org:10.1093/nar/gkac382>

6 Dobin, A. *et al.* STAR: Ultrafast universal RNA-seq aligner. *Bioinformatics* **29** (2013). <https://doi.org:10.1093/bioinformatics/bts635>

7 Frankish, A. *et al.* GENCODE 2021. *Nucleic Acids Res* **49**, D916-d923 (2021). <https://doi.org:10.1093/nar/gkaa1087>

8 Love, M. I., Huber, W. & Anders, S. Moderated estimation of fold change and dispersion for RNA-seq data with DESeq2. *Genome Biology* **15**, 550 (2014). <https://doi.org:10.1186/s13059-014-0550-8>

9 Wickham, H. *ggplot2: Elegant Graphics for Data Analysis. Second Edition. Springer*. Vol. 35 (2016).

10 Wickham, H. *ggplot2: Elegant Graphics for Data Analysis*. (Springer-Verlag, 2016).

11 Durinck, S. *et al.* BioMart and Bioconductor: a powerful link between biological databases and microarray data analysis. *Bioinformatics* **21**, 3439-3440 (2005). <https://doi.org:10.1093/bioinformatics/bti525>

12 Durinck, S., Spellman, P. T., Birney, E. & Huber, W. Mapping identifiers for the integration of genomic datasets with the R/Bioconductor package biomaRt. *Nat Protoc* **4**, 1184-1191 (2009). <https://doi.org:10.1038/nprot.2009.97>

13 Kamoun, A. *et al.* A Consensus Molecular Classification of Muscle-invasive Bladder Cancer. *Eur Urol* **77**, 420-433 (2020). <https://doi.org:10.1016/j.eururo.2019.09.006>

14 Lindskrog, S. V. *et al.* An integrated multi-omics analysis identifies prognostic molecular subtypes of non-muscle-invasive bladder cancer. *Nature Communications* **12**, 2301 (2021). <https://doi.org:10.1038/s41467-021-22465-w>

15 Racle, J. & Gfeller, D. EPIC: A Tool to Estimate the Proportions of Different Cell Types from Bulk Gene Expression Data. *Methods Mol Biol* **2120**, 233-248 (2020). <https://doi.org:10.1007/978-1-0716-0327-7_17>

16 Ritchie, M. E. *et al.* limma powers differential expression analyses for RNA-sequencing and microarray studies. *Nucleic Acids Res.* **43**, e47-e47 (2015). <https://doi.org:10.1093/nar/gkv007>

17 Stephens, M. False discovery rates: a new deal. *Biostatistics* **18**, 275-294 (2016). <https://doi.org:10.1093/biostatistics/kxw041>

18 msigdbr: MSigDB Gene Sets for Multiple Organisms in a Tidy Data Format (2020).

19 Wu, T. *et al.* clusterProfiler 4.0: A universal enrichment tool for interpreting omics data. *Innovation* **2**, 100141 (2021). <https://doi.org:10.1016/j.xinn.2021.100141>

20 BiocParallel: Bioconductor facilities for parallel evaluation (2021).

21 dplyr: A Grammar of Data Manipulation (2023).

22 Frappart, P. O. *et al.* Pancreatic cancer-derived organoids - a disease modeling tool to predict drug response. *United European Gastroenterol J* **8**, 594-606 (2020). <https://doi.org:10.1177/2050640620905183>
